# Dry Coupling of Ultrasonic Transducer Components for High Temperature Applications

**DOI:** 10.3390/s19245383

**Published:** 2019-12-06

**Authors:** Neelesh Bhadwal, Mina Torabi Milani, Thomas Coyle, Anthony Sinclair

**Affiliations:** 1 Department of Mechanical and Industrial Engineering, University of Toronto, Toronto, ON M5S 3G8, Canada; mtorabi@mie.utoronto.ca (M.T.M.); sinclair@mie.utoronto.ca (A.S.); 2 Department of Materials Science and Engineering, University of Toronto, Toronto, ON M5S 3E4, Canada; coyle@ecf.utoronto.ca

**Keywords:** ultrasonic transducer, elevated temperature, dry coupling, harsh environment, lithium niobate

## Abstract

The viability for dry coupling of piezoelectric ultrasonic transducer components was investigated, using a thin foil of annealed silver as a filler material/coupling agent at each component interface. Criteria used for room temperature evaluation were centered on signal-to-noise ratio (SNR) and echo bandwidth, for a Li-Nb based transducer operating in pulse-echo mode. A normal clamping stress of only 25 MPa, applied repeatedly over three loading cycles on a precisely-aligned transducer stack, was sufficient to yield backwall echoes with a SNR greater than 25 dB, and a 3 dB bandwidth of approximately 65%. This compares to a SNR of 32 dB and a 3 dB bandwidth of 65%, achievable when all transducer interfaces were coupled with ultrasonic gel. The respective roles of a soft filler material, alignment of transducer components, cyclic clamping, component roughness, and component flatness were evaluated in achieving this high efficiency dry coupling, with transducer clamping forces far lower than previously reported. Preliminary high temperature tests indicate that this coupling method is suitable for high temperature and achieves signal quality comparable to that at room temperature with ultrasonic gel.

## 1. Introduction

Ultrasonic transducers are used to inspect engineering components for flaws so that remedial action can be taken to avoid component failure. In some cases, it is desirable that the transducers be permanently mounted at critical piping/pressure vessel sites for continuous on-line monitoring of pipe integrity or characteristics of fluids inside the pipe. The pipe or pressure vessel may be very hot, such that the transducer must withstand high temperatures (and perhaps thermal cycling) for extended periods. Potential industrial applications include electrical power plants and petrochemical processing installations. Such a monitoring system reduces the need for plant shutdown to inspect the integrity of components and gives early warning of anomalous condition of the fluids inside the pipe or pressure vessel.

High-temperature ultrasonic inspection techniques in current use include the use of delay lines in front of the transducer to isolate the transducer from the high temperature [1,2]. Water cooling of the delay line or the transducer can also be used to increase the service temperature of traditional ultrasonic transducers by keeping the temperature of the ultrasonic transducer well below the temperature of the component being tested [2]. Electromagnetic acoustic transducers (EMAT) have been used up to 900 °C with active cooling, or 450 °C without active cooling, for continuous inspection [3,4]. EMATs; however, do suffer from high power needs, have poor signal-to-noise ratio, and are very sensitive to perturbations in sensor lift-off [5].

The overall long-term objective of this research project is to develop a direct-contact high-temperature ultrasonic transducer (continuous operation at up to 800 °C, with occasional thermal cycling) for non-destructive testing and process monitoring in the petrochemical industry. Currently, direct-contact non-destructive testing with ultrasonic transducers can only be done up to 550 °C without the use of cooling systems and delay lines [2,6]. Such commercially available high-temperature ultrasonic transducers for continuous use can cost several thousand dollars and are prone to failure after less than two years of use [5]. These transducers may fail due to a mixture of the following:

- Thermal stresses, causing cracking of components and breakdown of acoustic coupling between transducer components;
- Failure of individual components due to phase transitions or melting;
- Depoling of the piezoelectric element in the transducer by exceeding its Curie temperature.

Our previous studies focused on the selection and manufacturing of the backing, piezoelectric element, and matching layer for a cylindrically-shaped high-temperature ultrasonic transducer [5,7]. The suitable transducer components identified for high-temperature application were a rhombohedral 36° Y-cut lithium niobate piezoelectric crystal, a porous zirconia mechanical backing, and a stainless-steel 321 protective layer [5,7]. Methods for acoustically coupling the backing to the piezoelectric element were investigated in those earlier studies; a brazing system based on a silver-copper 72-28 braze (AgCu) was developed, but led to high reject rates of transducers due to cracking of the piezoelectric element [7,8].

**Figure 1:**
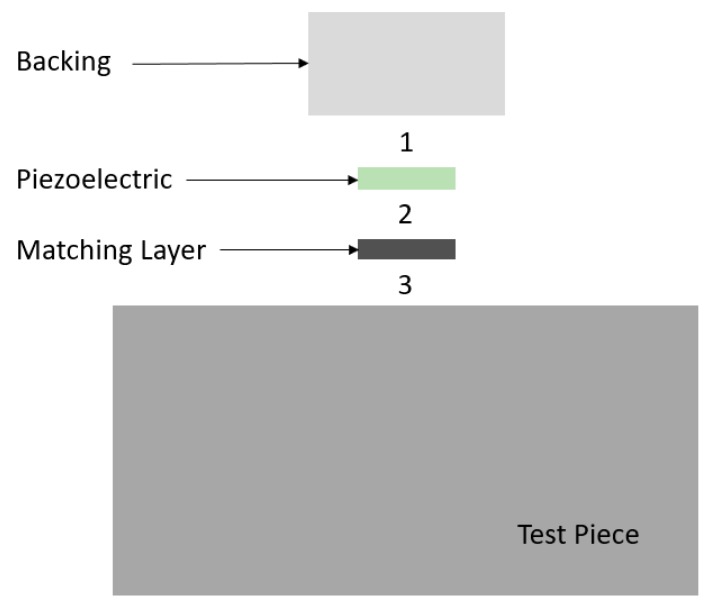
Transducer stack and interfaces.

The current study focuses on acoustically coupling the three principal transducer components together via dry coupling at each interface. Each pair of contacting materials must have similar acoustic impedance values for effective energy transmission. Air has an acoustic impedance value that is significantly lower than that of solid materials, such that any interfacial air gaps will block virtually all transmission of ultrasound from one transducer component to the next. Figure 1 shows the different components of the transducer stack and labels the three interfaces that were to be coupled. Interface (1)—backing–piezoelectric; interface (2)—piezoelectric–matching layer; and interface (3)—matching layer–test piece.

For dry ultrasonic coupling of any two components, high clamping pressure is applied to the two contacting surfaces; the goal is to cause one or both components to slightly deform at the interface, in order to mate with the other component and expel any air trapped between them. It is critical that these high clamping loads be distributed uniformly over the entire interface, to avoid the prospect of good ultrasonically coupling over only a limited portion of the nominal interfacial area. Nonuniformly distributed loads also create bending moments in the contacting parts, which may lead to failure in brittle components such as the piezoelectric element.

For the cylindrical components in our transducer, the following distinct factors are important to achieve uniform load distribution across the interfaces linking ultrasonic transducer components [9]:

- Specimen ends must be flat, parallel, and perpendicular to the lateral surfaces.
- Diameters of the individual components should be constant throughout the component.
- Components should be concentrically aligned.

Once uniform load distribution is obtained, the magnitude of dry coupling pressure across an interface required for efficient ultrasonic coupling is dependent primarily on (1) surface finish (roughness), (2) plastic deformation of asperities on each surface, and (3) material hardness [10,11].

*Surface finish:* Rough surfaces with small narrow bumps will require sufficient clamping forces to flatten out the peaks and valleys at the interface in order to expel any air gaps (i.e., there must be localized yielding and small-scale plastic flow of the protruding material). The required clamping force to achieve good coupling is; therefore, expected to be higher if one or both contacting surfaces are very rough. Although the average dry coupling pressure is the clamping force applied to the transducer components divided by the cross-sectional area of the components, the pressure at individual protruding locations can be much higher. Therefore, the pressure on the protrusions can surpass the material yield stress, even at relatively low transducer clamping loads.

*Plastic deformation of asperities:* During the compression loading of an interface, plastic deformation of the asperities takes places at loads that correspond to nominal pressures significantly below the yield strength of the material [10]. When the load is subsequently reduced, the plastic deformation remains such that the two surfaces are now better “matched” to each other. If the interface is then loaded again to the same level as before, primarily elastic deformation of the asperities takes place [10,12]. Lower loads are; therefore, required during the second or third loading cycle for the same degree of acoustic transmission through an interface.

*Material Hardness:* An interface between relatively soft materials require a lower clamping pressure for dry coupling pressure, as the softer materials deform more easily. For an interface between hard materials, a soft “filler” material, such as a gold or silver foil, can be placed between the contacting surfaces to bridge any gap caused by the roughness or waviness of either surface [13]. The clamping pressure required for dry coupling is, thereby, reduced.

Several researchers suggest that the dry coupling pressure required for acoustic coupling of two metallic surfaces is in the order of a few hundred megapascals [10,12,14,15,16]. Such high stresses can damage the piezoelectric element as well as permanently deform the test piece, if the stress linking transducer components is also placed on the pipe or pressure vessel under inspection; at this point ultrasound inspection is no longer non-destructive.

For these reasons, techniques to minimize the required pressure for dry coupling were investigated. The objective of this study was to determine the dry coupling pressures required for each interface of the transducer stack. The dry coupling pressure was to be reduced from expected values in literature using proper alignment, cyclic loading, and soft filler materials, so as to make it a feasible coupling method in high temperature transducers. For this initial investigation, tests and analysis are conducted at room and elevated temperature, such that dry coupling performance can be compared to that achievable with ultrasonic gel couplant.

## 2. Materials and Methods

The components of the high-temperature ultrasonic transducer design were:

- Rhombohedral 36° Y-cut lithium niobate piezoelectric crystal: The discs were designed to resonate at 3 MHz and were obtained from Boston Piezo Optics Inc. in Bellingham, MA, USA. The surface of the LiNbO3 was fine-lapped and bare of any electrode material. The crystal had a diameter of 1 cm and a thickness of 1 mm.
- Porous zirconia mechanical backing: A novel process to create porous ceramic backings was reported in previous work, in which polyethylene beads of diameter 75–90 microns were mixed into yttria stabilized powder [5,7,17]. The powder was then mixed and pressed in a die to form a “green” body. During the subsequent sintering process, the beads vaporized and left behind spherical cavities (pores). The backing was 1 cm thick with a diameter of 15 mm.
- Stainless-steel 321 protective layer: Square matching layers (1.5 × 1.5 cm) were cut from shim stock of thickness 0.41 mm [7].

A block of low carbon steel was used as the test piece.

### 2.1. Alignment

In order to determine the dry coupling pressure required for each interface of the ultrasonic transducer, the entire stack (backing, piezoelectric element, protective layer) was subjected to compression loading in a load frame at room temperature. ASTM E9: Standard Test Methods of Compression Testing of Metallic Materials at Room Temperature states the following requirements for samples undergoing compression tests [9]:

1. If possible, cylindrical test specimens should be used.
2. Specimen surfaces should have a surface roughness of 1.6 μm (63 μin) R_a_ or better.
3. Specimen ends must be flat and parallel 0.0005 mm/mm (in./in.) and perpendicular to the lateral surfaces to within 3’ of arc.
4. Diameter of the specimen should not vary more than 1% of the specimen length or 0.05 mm (0.002 in).
5. The centerlines of all lateral surfaces of the specimens shall be coaxial within 0.25 mm (0.01 in).

Although this standard is meant for metals and is not directly applicable to our ultrasonic transducer, it does indicate the key parameters for compression tests. The transducer components for our study had a deviation from parallelism of less than 0.002 mm/mm.

**Figure 2:**
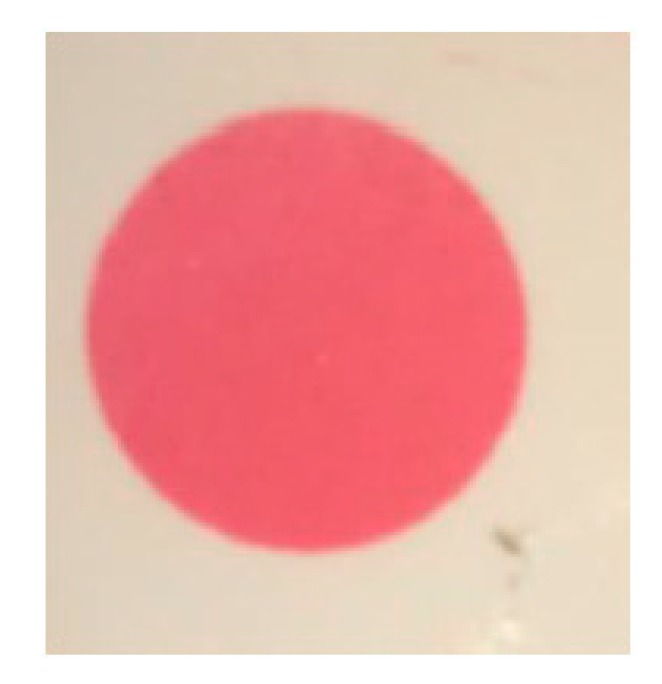
Uniform shade of pink on pressure film.

To qualitatively assess whether the pressure distribution on the lithium niobate crystal was uniform, the transducer stack (consisting of backing, crystal, matching layer) was placed on top of the low carbon steel test piece in an Instron 3367 load frame with a 30 kN load cell. A piece of Fuji Prescale^®^ Medium Film (obtained from Fujifilm Canada) was placed between the crystal surface matching layer. This film turns various shades of pink when pressures between 9.8 to 49.0 MPa (1400–7100 psi) are applied to it, according to charts provided by the manufacturer. The transducer stack was compressed, and a circle with a single shade of pink was formed on the film (Figure 2). Thus, a uniform load distribution was obtained, to within the manufacturer-specified uncertainty of ±10% [18].

### 2.2. Setup of Dry Coupling Tests

**Figure 3:**
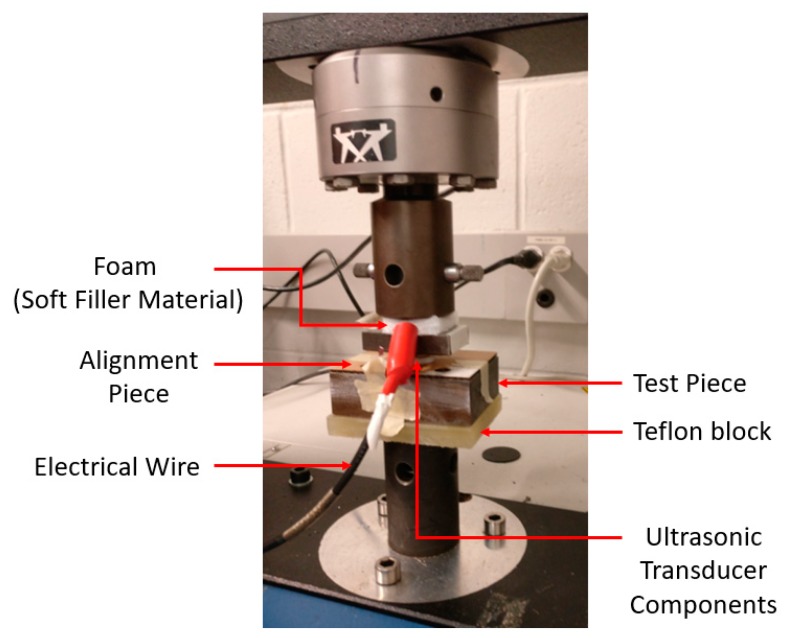
Load frame setup.

Further load frame tests were conducted to determine the dry coupling pressure required at each transducer interface if a filler material is used to achieve good signal transmission (Figure 3). Ultrasonic gel was inserted at two of the interfaces, while a filler material foil was placed in between the two components whose dry coupling pressure was to be determined. Surface characteristics of the transducer components are listed in Table 1, where R_a_ is the average roughness and W_a_ is the average waviness (W_a_) of the surface.

An Olympus 5077PR Square Wave Pulser/Receiver was used to excite the piezoelectric crystal with rectangular pulses of 300 V and a width of 17 μs. The pulses were sent into the test piece and an Agilent InfiniiVision DSO-X 2022A oscilloscope was then used to capture the backwall echo at each load increment of the load frame, for three loading cycles. A sampling frequency of 100 MHz was used, averaging 64 acquisitions per capture. MATLAB was then used to generate a normalized frequency spectrum using a Hann window with a length of 2 to 4 μs. It is important to note that the test piece was acoustically homogenous and thus no extra signal reconstruction was needed, as would be in the case of an acoustically heterogeneous material with attenuation distributions [19,20].

As shown in [10,12], repeated loading of the dry coupled ultrasonic transducer reduces the load required to obtain a specified level of acoustic transmission. As there was only a small change in acoustic transmission after the second loading cycle, it was decided that our ultrasonic transducer would be subjected to three loading cycles to reach the limiting case of plastic deformation of filler material and signal transmission across interfaces.

A filler material to be placed between the two components at each interface of the transducer was selected to meet the following criteria:

- Must be softer than components being coupled together;
- Chemically and physically stable in the presence of the transducer components;
- Must be an electrical conductor to act as electrodes for the lithium niobate crystal.

Transducers were mounted onto the test piece using ultrasonic gel; an investigation of transducer mounting techniques for various industrial applications is beyond the scope of this investigation.

Leading candidates for the filler material were gold and silver foil. Gold foil was more expensive and has an acoustic impedance of 62.6 MRayl, which was significantly higher than the transducer components (backing—20.7 MRayl [7], crystal—34.1 MRayl [21], matching layer—39.3 [22]) [23]. It was not expected that the filler material’s bulk properties (such as attenuation) would have a significant impact on the transmission of ultrasound through the filler, as the thickness of the material (50 μm) was significantly less than that of the ultrasonic wavelength.

Silver, which has an acoustic impedance of 37.8 MRayl, was chosen as the filler material [23]. Silver foil 50 μm thick was obtained and tested in both annealed and as-rolled form. Both types of silver foil had a purity greater than 99.95% [24,25]. As-rolled silver is roughly four times harder than annealed silver (Vickers Hardness) [26].

## 3. Results

### 3.1. Maximum Performance Test

As a reference point, “optimal coupling” was defined as the transducer performance when ultrasonic gel was placed between each interface of the stack; the backwall echo from the test piece was recorded for that reference configuration. The stack was loaded up to 24 kN, equivalent to a pressure of 305.7 MPa on the crystal. The echo SNR and bandwidth of the echo were largely independent of interfacial loading when gel coupling was used to link the transducer components:

- SNR ~32 dB
- Echo signal bandwidth ~65%

This optimal bandwidth figure was consistent with previous studies where a lithium niobate crystal was brazed onto the porous zirconia backing with AgCu braze foil [7,8].

### 3.2. As-Rolled Silver Foil Test

For interfacial pressure tests with as-rolled silver foil, transducer stacks were loaded in increments of 2 kN to a maximum load of 24 kN; the backwall echo signal was recorded at each load increment.

The best performance achievable using as-rolled silver foil as an interfacial couplant/filler material was an echo SNR of 25 dB and 60% bandwidth. The pressure needed on the entire stack was ~18 kN (equivalent to 229.3 MPa on the crystal). Table 2 shows the individual dry coupling loads required at each interface to achieve this target.

It is noted that all the crystals loaded up to 24 kN developed cracks that originated at the crystal periphery. Although these transducers were still functional, the cracks have negative implications for long term transducer performance, and no further tests were conducted with the as-rolled solver foil as a coupling agent. An alternative dry coupling system was required that involves a lower coupling load.

### 3.3. Annealed Silver Foil Test

Transducer loading trials were conducted using the experimental configuration described in Section 3.2, but using annealed silver as opposed to as-rolled silver foil at each transducer interface. The amplitude of the ultrasonic echo was continuously monitored as the axial load was increased. A cycling loading sequence was used: Once the echo amplitude had stabilized with increasing pressure on the transducer stack, the load was reduced to 0.5 kN, and the stack was then reloaded multiple times. The backwall ultrasonic echo was recorded at every kilonewton of load.

No cracks in the crystals were formed during these compression tests using annealed silver foil, as the dry coupling loads and associate pressure required to reach a stable echo amplitude were much lower than those required in the previous section using as-rolled silver foil. Table 3 shows the load required at each interface to achieve stable ultrasonic coupling, in the first and third loading cycles.

In comparison to the dry coupling loads required when as-rolled silver was used as the filler material, the loads required with annealed silver are ~2 to 3 times lower (see Table 2).

An instability was noted in the frequency spectra of the echoes for very low loading levels with the use of annealed foil. However, the echo shape and frequency spectrum stabilized once loaded to the point where good interfacial contact was achieved.

#### Annealed Silver Foil Test Piece

An investigation of transducer mounting techniques for various industrial applications is beyond the scope of this investigation, but could be an indicator for an area needing further investigation.

In the following experiments, annealed silver was placed inside all three interfaces of the transducer stack and the test piece. The low carbon steel test piece was hand polished with 100 grit sandpaper—the same surface preparation technique used by the project’s industrial sponsor to prepare the pipe surfaces on which ultrasonic transducers are mounted. The axial compressive force exerted by the load frame was increased in increments; the SNR and peak-to-peak voltage of the backwall echo signal were recorded at each load increment. The test was stopped when the peak-to-peak voltage stabilized. The load was then reduced to 0.5 kN and reapplied.

**Figure 4:**
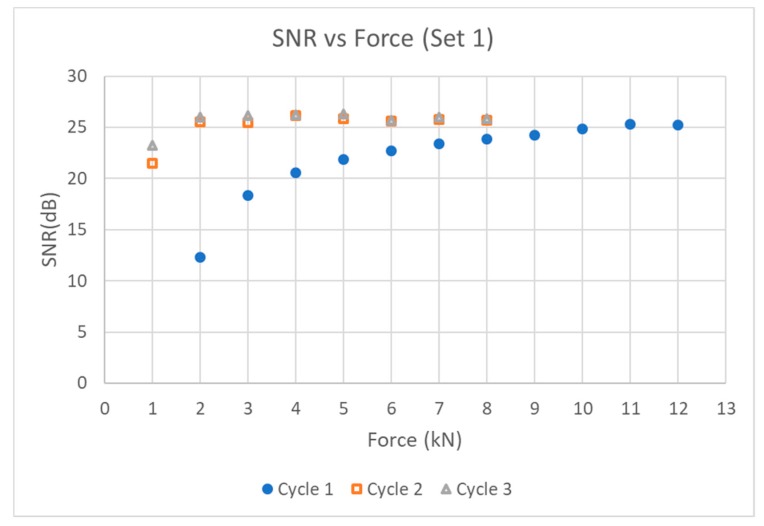
Axial loading for Set 1.

**Figure 5:**
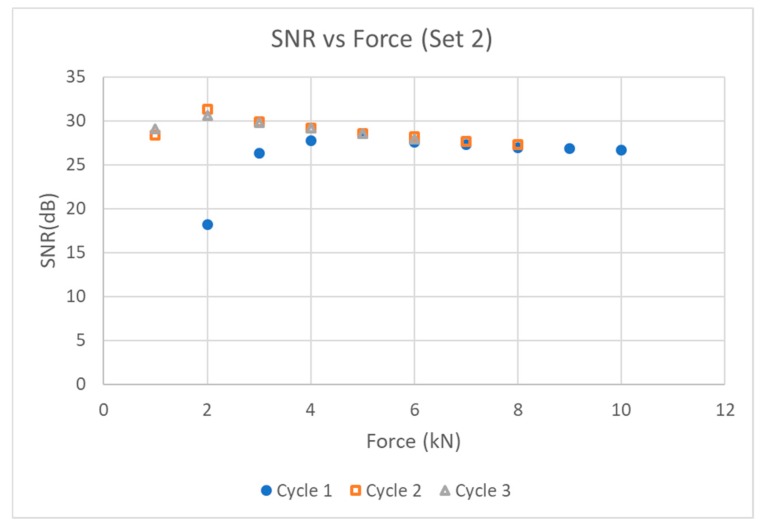
Axial loading for Set 2.

Figure 4 and Figure 5 show the SNR vs force graphs for two different sets of transducer components Sets 1 and 2. By the third loading cycle, a load of only 2 kN (25.5 MPa) was enough to produce an echo that had stabilized with a maximum SNR near 65% in both samples, with a spectrum centered at 3 MHz.

It is noted that the load required to achieve stable coupling during the first loading cycle was different for Set 1 and Set 2. Additionally, the SNR achieved in Set 2 was larger than that obtained in Set 1. This may be due to the roughness of test piece 1 being pressed down and flattened after loading with the first transducer—a phenomenon reported in literature [10,12]. This effect is prominent for test pieces with larger air gaps in the roughness of the surface.

In summary, a rough, carbon steel surface typical of that found in an industrial plant can be acoustically coupled to our transducer prototype after cyclic loading three times with a load of 2 kN. When practical, pre-pressing of the surface can “flatten” surface roughness to a certain degree, making efficient dry coupling with annealed silver foil achievable at even lower loads.

### 3.4. High Temperature Coupling Test

**Figure 6:**
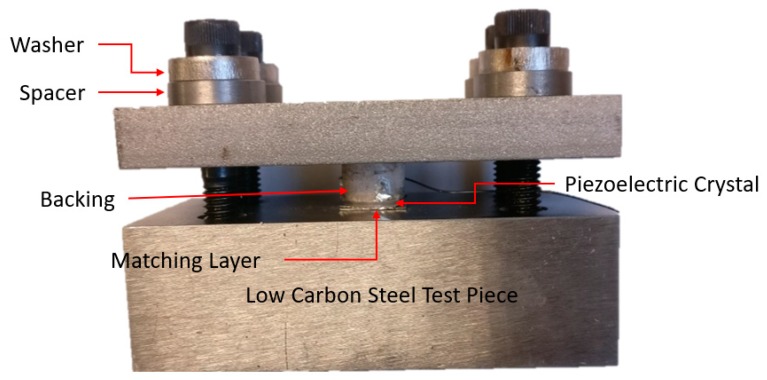
High temperature test fixture.

A high temperature test was carried out to determine the suitability of this dry coupling method. Figure 6 shows a transducer assembly—a backing, piezoelectric crystal, and matching layer—coupled to a ground low carbon steel test piece. Annealed silver foil was placed in between the three interfaces (see Figure 1). The fixture was placed in the load frame and loaded up to 12 kN (152.9 MPa), unloaded, and then loaded to 8 kN (101.9 MPa). It was then unloaded and four screws with disc springs were tightened to a load of 3 kN (38.2 MPa). The disc springs were used to compensate for thermal expansion during the high temperature test. Spacers were machined to an exact height, so that when the screws were tightened, the washer on each bolt would touch the spacer when the springs provided a combined force of 3 kN on the transducer stack.

**Figure 7:**
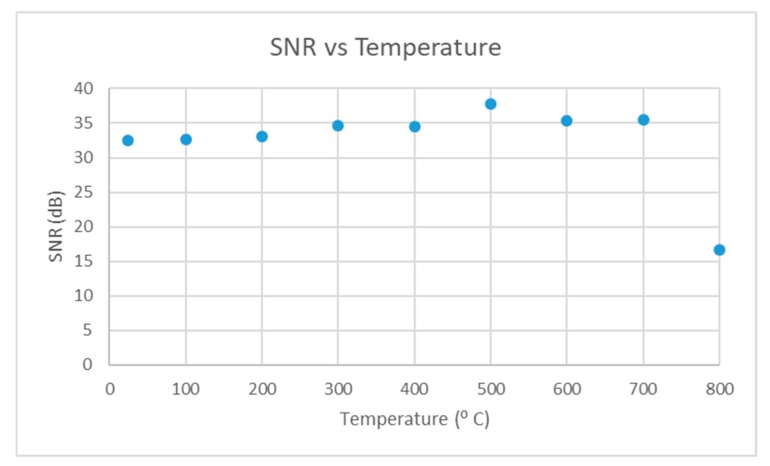
SNR vs temperature.

**Figure 8:**
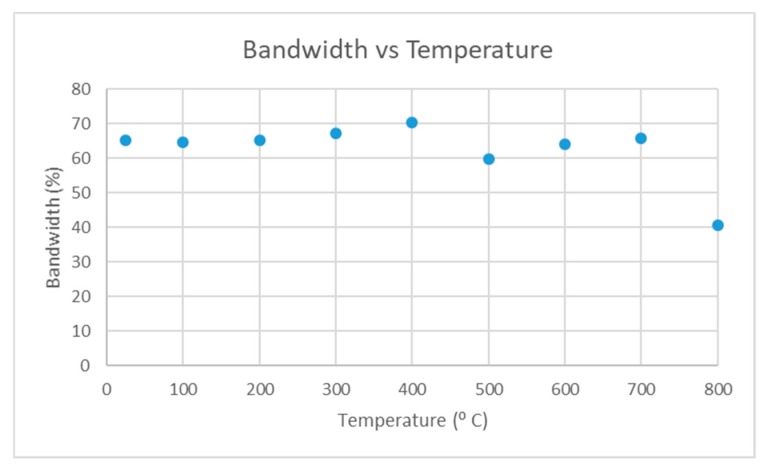
Bandwidth vs temperature.

The fixture was placed in a furnace and heated up to 800 °C at 4 °C/min. The echo from the test piece was recorded at room temperature and every 100 °C. The SNR and bandwidth of the echo at each temperature increment are plotted in Figure 7 and Figure 8.

The SNR of the echo started at 32 dB and increased until 700 °C, after which there was a large drop. The bandwidth of the echo remained between 60% to 70% until 700 °C, after which there was a large drop. When the fixture was cooled and removed from the furnace it was noted that the screws were loose. The disc springs may have deteriorated at higher temperatures (their service temperature was 600 °C) and this could have led to a loss in dry coupling pressure resulting in the drop in signal quality at 800 °C. It is important to note that after the high temperature test was conducted the annealed silver foil was inspected and no signs of deterioration were observed.

To summarize the preliminary high temperature test suggests that this dry coupling method is suitable for high temperature applications, as the signal quality is comparable to that at room temperature with ultrasonic gel.

## 4. Discussion

The objective of this study was to determine the dry coupling pressures required for each interface of the transducer stack. Important criteria for proper transducer alignment were identified and a uniform load distribution was obtained when axially loading the transducer.

Two potential filler materials were identified to be placed between the component surfaces of the transducer stack as-rolled silver foil, and annealed silver foil.

With as-rolled silver foil, the dry coupling pressure required to produce an echo with an SNR of 25 dB and 60% bandwidth was 18 kN (229.3 MPa). Cracks were formed in the crystal during loading to 24 kN and thus further testing with cyclic loading was not carried out. Although these transducers were still functional, the cracks have negative implications for long term transducer performance and the dry coupling pressure was to be lowered.

The use of annealed silver foil reduced the dry coupling pressure required to couple the transducer layers together. The dry coupling pressure required between the backing and the crystal interface dropped by a factor of three and the dry coupling pressure required between the crystal and matching layer dropped by a factor of two. This is consistent with literature where drying coupling pressures depends on material hardness [11,13].

Cyclic loading of the transducer stack three times after initial assembly, with annealed foil as the filler material, reduced further the load required to 1 kN (12.7 MPa) in both interfaces. Cyclic loading caused the of surface deviations of the interfaces to be pressed down and flattened to a certain degree. Cyclic loading also caused the two surfaces to be better “matched” to each other, as shown in literature [10,12].

The few hundred megapascals dry coupling pressure suggested for acoustic coupling of two metallic interfaces can be greatly reduced through the use of precision aligned transducers, soft filler materials, and cyclic loading. Preliminary high temperature tests suggest that this method is suitable for high temperatures. Signals at high temperatures were comparable to that at room temperature. This may make dry coupling a feasible coupling method in high temperature transducers and may be an answer to a major problem in high temperature transducer manufacture.

For this initial investigation, tests and analysis are conducted at room and elevated temperatures, such that dry coupling performance can be compared to that achievable with ultrasonic gel couplant. Future work may be focused on the dry coupling of the transducer components with annealed silver or other filler materials with suitable properties at high temperatures. Future high temperature tests may include thermal cycling and filler material durability testing. Devising a scheme to keep constant pressure up to 800 °C will also be a target for future work.

## Figures and Tables

**Table 1:** Surface properties.

Component	Roughness (R_a_) nm	Waviness (W_a_) nm
**Lithium Niobate**	174	7
**Matching Layer**	198	1356
**Test Piece (100 grit sandpaper)**	798	1255

**Table 2:** Dry coupling loads (as-rolled silver).

Interface	Load (kN)	Pressure on Crystal (MPa)
(1) Backing–Crystal	18	229.3
(2) Crystal–Matching Layer	6	76.4

**Table 3:** Dry coupling loads (annealed silver).

Interface	Load (kN) During 1st Loading Cycle	Pressure on Crystal (MPa)	Load (kN) During 3rd Loading Cycle	Pressure on Crystal (MPa)
(1) Backing–Crystal	6	76.4	1	12.7
(2) Crystal–Matching Layer	3	38.2	1	12.7
